# Health-Related Quality of Life in People with Advanced HIV Disease, from 1996 to 2021: Systematic Review and Meta-analysis

**DOI:** 10.1007/s10461-024-04298-y

**Published:** 2024-05-14

**Authors:** I. Portilla-Tamarit, M. Rubio-Aparicio, M. J. Fuster-RuizdeApodaca, J. Portilla-Tamarit, S. Reus, J. Portilla

**Affiliations:** 1 Department of Health Psychology, University of Alicante, Alicante, Spain; 2 Alicante Institute for Health and Biomedical Research (ISABIAL), Alicante, Spain; 3 Infectious Diseases Unit, Department of Internal Medicine, Alicante University General Hospital, Alicante, Spain; 4 Spanish AIDS Research Network, Carlos III Health Institute, Madrid, Spain; 5 Department of Basic Psychology & Methodology, Faculty of Psychology and Speech Therapy, University of Murcia, Avda. Teniente Flomesta, 5, 30003 Murcia, Spain; 6 Faculty of Psychology, National Distance Learning University (UNED), Madrid, Spain; 7 Spanish Interdisciplinary AIDS Society (SEISIDA), 28036 Madrid, Spain; 8 Department of Clinical Medicine, Miguel Hernandez University, Elche, Alicante Spain

**Keywords:** Advanced HIV disease, Health related quality of life, Systematic review, Meta-analysis, HIV, AIDS, Low CD4, Enfermedad avanzada de sida, Calidad de vida realacionada con la salud, Revision sistemática, Meta-analisis, VIH, Sida, Bajos CD4

## Abstract

The purpose of the study was to assess the effects of advanced HIV disease (AHD) on health-related quality of life (HRQoL) in PLHIV, the changes in HRQoL outcomes over the last 25 years, and the differences between countries according to level of economic development. We conducted a systematic review and meta-analysis. The search was conducted in PubMed and Web of Science using the terms: “health-related quality of life”, “HQRoL”, “HIV”, “AIDS”, “advanced HIV disease” and “low CD4 cells”. Studies inclusion criteria were: adult population; initiated after 1996 and published before July 2021; clinical trials, cross-sectional, cohort, and case–control studies; studies analyzing the relationship between AHD and HRQoL; English or Spanish language. Standardized mean differences (*d*+) were calculated to estimate the effect size for the meta-analyses. Summary statistics were calculated using a random-effects model, and analyses of effect moderators, using mixed-effects models. The meta-analysis included 38 studies. The results indicated that HRQoL is worse in patients with AHD compared to those without. The main HRQoL domains affected were *overall health perception and concern* and *physical and functional health and symptoms.* We found a moderate impact for age and gender on some HRQoL domains. There were no differences in relation to socioeconomic inequities, country of residence, or time period analyzed. In conclusion, advanced HIV disease has a negative impact on health and well-being in PLHIV. Our results show that despite all the advances in antiretroviral treatments over the last 25 years, AHD persists as a source of extreme vulnerability, regardless of where PLHIV live.

**Supplementary Information:**

The online version contains supplementary material available at 10.1007/s10461-024-04298-y.

## Resumen

El objetivo del estudio fue evaluar los efectos de la enfermedad avanzada de sida (EAS) en la calidad de vida relacionada con la salud (CVRS) en personas que viven con el VIH (PVVIH), los cambios experimentados en la CVRS en los últimos 25 años y las diferencias entre países. Realizamos una revisión sistemática y metaanálisis. La búsqueda se llevó a cabo en PubMed y Web of Science utilizando los términos: “calidad de vida relacionada con la salud”, “CVRS”, “VIH”, “SIDA”, “enfermedad avanzada por VIH” y “células CD4 bajas”. Los criterios de inclusión de los estudios fueron: población adulta; iniciado después de 1996 y publicado antes de julio de 2021; ensayos clínicos, estudios transversales, de cohorte y de casos y controles; estudios que analizan la relación entre EAS y CVRS; idioma inglés o español. Se calcularon diferencias de medias estandarizadas (*d*+) para estimar el tamaño del efecto para los metaanálisis. Los efectos promedios se calcularon utilizando un modelo de efectos aleatorios, y el análisis de moderadores utilizando modelos de efectos mixtos. El metaanálisis incluyó 38 estudios. Los resultados indicaron que la CVRS es peor en pacientes con EAS en comparación con aquellos sin EAS. Los principales dominios de CVRS afectados son la *percepción de salud general y su preocupación*, y la *función física y de salud y los síntomas asociados*. Encontramos un impacto moderado por edad y género en algunos dominios de CVRS. No encontramos diferencias en cuanto a las desigualdades socioeconómicas, país de residencia o período de tiempo analizado. En conclusión, la enfermedad avanzada por VIH tiene un impacto negativo en la salud y el bienestar en las personas con VIH. Nuestros resultados muestran que, a pesar de todos los avances en los tratamientos antirretrovirales en los últimos 25 años, el EAS persiste como una fuente de extrema vulnerabilidad, independientemente de dónde vivan las personas con VIH.

## Introduction

In 1996, the advent of combined active antiretroviral therapy (cART) dramatically transformed the face of AIDS [1]. Since then, the efficacy and safety of antiretroviral therapies have improved, while AIDS-related morbidity and mortality have continued to decrease [1]. In 2014, the World Health Organization (WHO) approved a new goal on HIV and AIDS: “*to end the AIDS epidemic as a public health threat by 2030*”. To achieve this objective, UNAIDS established the “90-90-90” targets, defined as: “90% of people living with HIV (PLHIV) will know their HIV status, 90% of people who know their HIV-positive status will be accessing treatment, and 90% of PLHIV on treatment will have suppressed viral loads.” This strategy promoted healthy lives and well-being for all PLHIV, regardless of their age or country of residence [2, 3]. Recently, these objectives have become more ambitious, raising the bar to ensure that 95% of PLHIV achieve these targets [4]. Since then, different authors have highlighted the importance of health-related quality of life (HRQoL) for attaining long-term health and well-being in PLHIV, calling on health systems, international societies, and others providing AIDS services to include it as an essential goal of HIV care [5, 6].

As a multidimensional concept, HRQoL can be influenced by many factors in PLHIV, including ageing, gender, HIV-related symptoms, comorbidities, substance abuse, depression, socioeconomic inequalities, employment, and financial stress, among others [7–13]. Understanding all the factors associated with HRQoL is critical for designing interventions to support PLHIV, especially vulnerable populations such as those accessing services with advanced HIV disease (AHD).

The WHO defines AHD as a CD4 + count under 200 cells/µL or a WHO clinical stage 3 or 4 [14]. Despite all the efforts to rapidly diagnose HIV infection, diagnosis of AHD is still frequent in PLHIV even in middle- and high-income countries. In 2021, of the 692,000 people in the European Union/European Economic Area who received a new HIV diagnosis, 35% were diagnosed with AHD [15]. Worldwide, about 650,000 people died from AIDS-related diseases in 2021, over half in the WHO Africa and Asia–Pacific regions [16].

AHD has a negative impact on the health of PLHIV [17, 18], increasing the likelihood of developing AIDS- and non-AIDS-defining disease, especially cancer, cardiovascular disease, kidney disease, and neurocognitive impairment. All these risks predispose those with AHD to experience more physical symptoms, including chronic pain, extreme fatigue, and a significant decline in their ability to carry out activities of daily living [19–22]. Moreover, AHD is associated with an increased risk of mortality and reduced life expectancy [21, 23]. Managing multiple medical conditions simultaneously can be overwhelming, negatively impacting emotional and psychological quality of life [24]. Furthermore, the stigma and discrimination associated with HIV may intensify in this advanced stage, leading to social isolation, self-esteem issues, and difficulties in accessing adequate medical care [25]. All these aspects suggest that AHD has a devastating impact on the physical, mental, and emotional health in PLHIV, ultimately affecting HRQoL.

However, there is no consensus on the impact of AHD on HRQoL. Different authors have reported that PLHIV with low CD4 counts or an AIDS-defining event have worse HRQoL than those with higher CD4 counts or without AIDS. However, there is no agreement about which HRQoL domains are most affected in people with AHD. Regarding CD4 + cells counts, studies by Fuster-RuizdeApodaca et al., Venturi et al., and Aden et al. have reported that lower CD4 counts correspond to lower overall HRQoL scores [26–28]. Emuren et al. and Liu et al. described that CD4 + cell counts below 200/µL were associated with worse physical but not mental health [29, 30]. On the other hand, Fumaz et al., focusing on gender differences, observed that poorer mental health in women was associated with lower CD4 cell counts but not with physical health [31]. Préau et al. reported similar results in a mixed-gender sample, finding a negative association between low CD4 cells and mental HRQoL [32]. Regarding AIDS, Emuren et al. and Préau et al. found that an AIDS diagnosis was linked with worse physical health but not with the mental health dimension [29, 33], while Fumaz et al. reported that AIDS-defining events were similarly associated with unacceptably low physical and mental HRQoL, regardless of gender [31]. Nevertheless, Badia et al. did not find any relationship between low CD4 counts or AIDS with lower HRQoL scoring [34]. The variability of these findings in different settings supports the need to synthesize all available evidence to clarify how AHD affects HRQoL.

Another aspect that warrants investigation is the effect of improvements in antiretroviral therapy (ART) over time. Since its introduction in 1996, ART has made gradual but steady advances in terms of better efficacy, decreased toxicity, and improved convenience. In the first years of the AIDS pandemic, surviving to an AHD diagnosis was the main challenge for PLHIV. In the mid-1990s, treatments with protease inhibitors (PIs) like ritonavir and indinavir plus zidovudine represented a significant breakthrough in the approach to HIV/AIDS, providing an opportunity for people with AHD [1]. The first PIs were associated with diarrhea, metabolic side effects, and lipodystrophy [35], while zidovudine, the first nucleoside retrotranscriptase inhibitor (NRTI), was associated with anemia and bone marrow suppression [36]. Other first-generation NRTIs, such as stavudine, zalcitabine, and didanosine were associated with severe mitochondrial toxicity, peripheral neuropathy, and pancreatitis [37]. Efavirenz, the first non-nucleoside retrotranscriptase inhibitor (NNRTI), and nevirapine were approved at the end of the twentieth century. Efavirenz was associated with neurotoxicity, while nevirapine could cause severe hypersensitivity hepatitis [38, 39]. In the 2000s, the second-generation therapies appeared, including boosted PI, new NRTI, and NNRTI, with greater efficacy, tolerance, and convenience [40]. These new drugs permitted the development of fixed-dose combination (FDC) therapies, reducing the pill burden and improving adherence. In this second period, PIs were associated with a higher cardiovascular risk [41], and tenofovir disoproxil-fumarate (TDF) with renal and bone toxicity [42]. In the mid-2000s, raltegravir, the first integrase strand-transfer inhibitor (INSTI), was approved for the treatment of HIV infection, followed by elvitegravir, dolutegravir, and bictegravir. The introduction of the latest INSTIs marked the third period of antiretroviral treatment, characterized by the widespread use of FDC in therapies and the development of potent bi-therapies for treating HIV infection. INSTIs have been associated with neuropsychiatric toxicity and obesity, but these adverse effects rarely lead to treatment discontinuation [43]. The success of ART has permitted the establishment of new strategies, such as early treatment of HIV infection regardless of the number of CD4 cells and the “test and treat” strategy, initiating ART as soon as possible [44, 45]. These extraordinary changes could support the hypothesis that improvements in ART from 1996 to 2021 have impacted HRQoL even in PLHIV with AHD. Unfortunately, access to remarkable advances in antiretroviral therapy has been disparate worldwide due to the varying availability and allocation of economic resources in different countries [46].

The aims of this meta-analysis are: (1) to assess the effects of AHD on HRQoL in PLHIV; (2) to analyze the probable changes on HRQoL outcomes over the last 25 years, from the advent of the first cART with PI to the new regimens of ART that use INSTI; (3) to assess heterogeneity among studies; and (4) to evaluate potential differences in HRQoL in PLHIV with AHD from different countries and levels of economic development: low-, middle- and high- income countries, and to examine the influence of other potential variables.

## Methods

### Protocol and Registration

The study protocol was pre-registered on the Open Science Framework (see 10.17605/OSF.IO/RZAMQ for further information). Furthermore, this systematic review and meta-analysis was conducted according to the Preferred Reporting Items for Systematic reviews and Meta-Analyses (PRISMA guidelines) [47]. Supplementary Table 1 presents the PRISMA checklist.

### Study Eligibility Criteria

Our study population was adults with AHD defined as AIDS (CDC clinical stage C3 or WHO HIV-clinical stage 3/4), or CD4 < 200 cells/µL [14]. Inclusion criteria were: (1) studies in PLHIV aged 18 years or older; (2) studies initiated after the advent of cART in 1996, and published before 31 July 2021; (3) clinical trials, cross-sectional, cohort, and case–control studies that included any HRQoL intervention; (4) studies analyzing the relationship between AHD and HRQoL, comparing HRQoL with versus without AIDS and/or with CD4 + lymphocytes ≥ 200 cells/μL versus < 200 cells/μL; (5) studies written in English or Spanish. Exclusion criteria were: (1) meta-analyses, systematic reviews, and secondary studies; (2) abstracts, conference proceedings, books or book chapters, unpublished material, and reports that were not peer-reviewed; (3) articles reporting HRQoL measures obtained using a non-validated questionnaire; (4) articles reporting lymphocyte CD4 + counts, not categorized as having more or less than 200 CD4 + cells/μL.

### Search Strategy

The search was conducted from 1 to 31 July 2021 in PubMed and the Web of Science. The following keywords were used: “health-related quality of life” [OR] “HQRoL” AND “HIV” [OR] “AIDS” [OR] “advanced HIV disease” [OR] “low CD4 cells”. Supplementary Table 2 presents the full search strategy. Additionally, the reference lists of the selected articles were handsearched to identify other potentially eligible studies.

Search results were de-duplicated, and four review authors (IPT, JP, JPT and SR) independently screened the titles and abstracts against the selection criteria. All relevant or potentially relevant records were retrieved for full-text review, which was also performed independently by IPT, JP, JPT, and SR. Disagreements were resolved by discussion between the four review authors. Doubts about HRQoL measures or meta-analysis methods were resolved by MJF, an expert in HRQoL, and MRA, an expert in meta-analyses.

### Data Extraction and Quality Assessment

Study characteristics were independently extracted by the researchers according to a predefined protocol and categorized based on article characteristics. The following information was collected: first author, publication year, country, World Bank income level, sample size, demographic characteristics of the sample (i.e., age, sex), study design, HRQoL questionnaires, recruitment period, percentage of patients on ART, percentage of patients with undetectable viral load, percentage of patients with CD4 +  < 200 cells/µL, and percentage of patients with AIDS (Table 1). Information was also extracted on study funding, authors’ conflict of interests, and other ethical issues.

**Table 1:** Characteristics of included studies

Study ID	Country	Country income level^a^	N	% men	Mean age (SD)	Study design	HQRoL Questionnaire^b^	Recruit-ment period	% participants with HIV-related characteristics			
									cART	undetectable VL	CD4 + < 200	AIDS
Ahmed et al. (2021a)	Pakistan	LMI	182	74	–	CS	WHOQOL-HIV BREF	2019	100	64.8	17.6	0
Ahmed et al. (2021)	Pakistan	LMI	602	65	–	CS	EQ-5D-3L	2019	100	51.7	31.6	3.3
Amara et al. (2020)	USA	HI	1587	77	43 (8.7)	Co	MOS-HIV	2003–07	63.5	–	59.3	–
Anis et al. (2009)	Several^c^	HI	368	98	48 (8.5)	CT	EQ-5D-5L & MOS-HIV	2001–07	100	–	–	26.6
Armon and Lichtenstein (2012)	USA	HI	159	91	42.67 (9.7)	CS	MOS-HIV	2006–07	92.5	84.3	–	62.1
Bekele et al. (2013)	Canada	HI	602	75	43.1 (8.6)	Co	MOS-HIV	–	74	–	–	50
Belay et al. (2011)	Ethiopia	LI	511	40	42 (11)	CS	EQ-5D-5L	2019	100	86.1	20	71.5
Burgoyne and Saunders (2001)	Canada	HI	113	87	38	CS	MOS-SF-36	2001	48.1	25	–	32
Call et al. (2000)	UK	HI	158	87	39 (8.6)	Co	MOS-SF-36	1997–99	25	0	61.4	–
Degroote et al. (2013)	Belgium	HI	237	75	45.8 (10.7)	CS	MOS-HIV	2012	92	74.3	2.5	–
Emuren et al. (2017)	USA	HI	1668	93	40	Co	MOS-SF-36	2006–10	100	49.4	6.1	11.5
Fuster-RuizdeApodaca et al. (2019)	Spain	HI	1462	79	45 (10.2)	CS	WHOQOL-HIV BREF	2016–17	100	90.4	4.8	–
Garcia-Ordoñez et al. (2001)	Spain	HI	300	76	33.9 (6.1)	C–C	MOS-SF-36	1997–98	–	–	45.3	31
Gibson et al. (2011)	Canada	HI	758	82	47 (10)	Co	MOS-SF-36	2006–09	–	74	–	26
Hailu et al. (2020)	Ethiopia	LI	577	40	37 (8.5)	CS	WHOQOL-HIV BREF	2019	100	–	–	25.8
Hays et al. (2000)	USA	HI	2864	77	39 (9)	Co	MOS-SF-36	1996	–	–	53	38
Igumbor et al. (2013a)	S Africa	UMI	311	41	39.3 (9.8)	Co	WHOQOL-HIV	–	0	–	91	–
Igumbor et al. (2013b)	S Africa	UMI	331	35	40.8 (9.7)	Co	WHOQOL-HIV	–	0	–	19.2	–
Imam et al. (2012)	Bangladesh	LMI	82	57	34.83 (7.4)	Cs	WHOQOL-HIV BREF	2009	–	–	–	34.2
Kanu and Tobin-West (2018)	Niger	LI	288	46	35.9 (10.7)	CS	WHOQOL-HIV BREF	–	100	–	–	50
Liping et al. (2015)	China	UMI	403	72	39.6 (12.2)	CS	WHOQOL-HIV BREF	2014	70.9	–	16.1	11.9
Mafirakureva et al. (2016)	Zimbabwe	LMI	257	28	39.7 (8.9)	CS	EQ-5D-3L & HAT-QoL	2013	100	–	23.4	42.8
Meemon et al. (2016)	Thailand	UMI	329	44	41.95 (7.8)	CS	WHOQOL-HIV BREF	2014	98	–	11.6	50.8
Melaku et al. (2020)	Ethiopia	LI	160	37	41.47 (9.5)	CS	PROQol-HIV	2016	100	–	60.6	63.7
Murri et al. (2015)	Italy	HI	809	68	36	Co	MOS-HIV	1997–98	89.9	32.1	38.1	32.5
Patel et al. (2017)	Kenya	LMI	538	35	–	CT	SF6D	2007–09	0	–	67.7	4.1
Peltzer and Phaswana-Mafuya (2008)	S Africa	UMI	607	22	–	CS	WHOQOL-HIV BREF	2007	48.1	–	32.3	66.4
Preau et al. (2007)	France	HI	72	67	41 (10)	CS	WHOQOL-HIV BREF	2004	90.0	60	11	21
Remor (2003)	Spain	HI	100	59	37.3 (8.3)	CS	MOS-SF-30	1999	91	–	–	37
Rueda et al. (2011)	Canada	HI	361	94	42 (9)	Co	MOS-HIV	–	–	55	–	63
Schnall et al. (2017)	USA	HI	1306	71	48.5 (11.7)	L	PROMIS-29	2016	–	–	–	35.7
Stasinopoulou et al. (2010)	Greece	HI	154	77	42.6 (9.4)	CS	MOS-HIV	–	–	–	13.6	24.7
Torres et al. (2018)	Several^d^	UMI, LMI, LI	512	49	39	CT	ACTG SF-21	2012–13	100	0	62	29
Tran (2012)	Vietnam	LMI	1016	64	35.4 (7)	CS	WHOQOL-HIV BREF	2012	88.8	–	31.1	37.6
Tran et al. (2012)	Vietnam	LMI	1016	64	35.4 (7)	CS	EQ-5D-5L	2012	88.8	–	24.5	37.6
Uchechukwu et al. (2020)	Niger	LI	969	65	35.47 (7.1)	CS	PROQol-HIV	2019	100	–	32.5	–
Venturini et al. (2017)	Guyana	UMI	943	66	50.9 (9.3)	CS	EQ-5D-3L	2015	–	74.2	6.7	–
Worthington and Krentz (2005)	Canada	HI	308	91	41 (8)	CT	MOS-HIV	2003	82	–	–	25
Zuñiga et al. (2011)	Mexico	UMI	239	84	36.9 (9.8)	CS	MOS-HIV	2001–04	–	–	29	29

*cART* combined antiretroviral treatment, *CS* cross-sectional, *CT* clinical trial, *Co* cohort, *L* longitudinal, *C*–*C* case–control, *SD* standard deviation, *VL* viral load

Ahmed et al. (2011a), Ahmed et al. (2021b), Ahmed et al. (2011b), Ahmed et al. (2021)

^a^World Bank classifications include low-income (LI), lower-middle income (LMI); upper-middle income (UMI); high income (HI)

^b^Generic HRQoL questionnaires: EQ-5D-5L, EQ-5D-3L, MOS-SF-36, MOS-SF-30, HAT-QoL, PROMIS-29, ACTG SF-21, SF6D; Questionnaires specific to PLHIV: MOS-HIV, PROQol-HIV, WHOQOL-HIV, WHOQOL-HIV BREF

^c^USA, UK, Canada

^d^Brazil, India, Kenya, Malawi, Peru, S Africa, Tanzania, Thailand and Zimbabwe

The methodological quality of the studies included in the meta-analysis was assessed with the Newcastle–Ottawa Scale (NOS), adapted for cross-sectional studies [48]. This scale uses a “star system” to judge quality based on three dimensions: selection, comparability, and outcome. The NOS consists of 7 items, and the total maximum quality score is 9 stars. Study quality was classified as “high” (8–9 stars), “moderate” (5–7 stars) or “low” (≤ 4 stars).

To assess the reliability of the data extraction, all studies were independently coded by two review authors (IPT and MRA), and inconsistencies were resolved by consensus. For categorical variables, kappa coefficients ranged between 0.810 and 1.0 (M = 0.931), and for continuous variables all intra-class correlations were equal to 1.0.

### Computation of Effect Sizes

Usually, HRQoL is measured as a multidimensional concept; however, not all HRQoL questionnaires have exactly the same dimensions. To make the comparison feasible, we categorized the most frequent dimensions according to similarity or by grouping the instruments containing summary indexes. Three review authors (PI, RM, MJF) carried out this process, discussing and resolving discrepancies by consensus. Six outcome domains were finally created: *overall health perception and concern*, *physical and functional health and symptoms*, *psychological health*, *social relationships*, *mental health summary*, and *physical health summary*. Supplementary Table 3 provides details on how dimensions of the questionnaires were categorized.

Once the six HQRoL domains were summarized, the effect sizes for each individual study were calculated. The effect size index was the standardized mean difference (*d*), defined as the difference between the mean of the treatment group and the mean of the control group, divided by a pooled standard deviation (SD): *d* = c(m)(yT − yC)∕SD, with c(m) representing a correction factor for small sample sizes [49]. In this meta-analysis, the *d* index was calculated to compare the differences in HRQoL between people diagnosed versus not diagnosed with AIDS, and between those with CD4 counts under versus over 200 cells/µL. In any case, the mean values of the non-AIDS and the CD4 ≥ 200 cells/µL groups (control groups) were subtracted from the means for the AIDS and the CD4 < 200 cells/µL groups (treatment groups), respectively, such that negative *d* values indicate poorer HRQoL in the AIDS and CD4 < 200 cells/µL groups. For standardization, absolute values of *d* of around 0.2, 0.5, and 0.8 were interpreted as small, moderate, and large magnitudes of effect, respectively [50]. For effect size calculations, the available means and SDs for each group were used. When this information was not reported, the corresponding authors were contacted to request the required values. In some studies, when the results were reported by means of odds ratio or correlation, conversion formulas were applied to obtain the corresponding *d* value [51, 52].

### Statistical Analysis

Separate meta-analyses were carried out with the effect sizes for each of the six outcome measures, for the comparison of the AIDS versus non-AIDS groups, and for the comparison of the CD4 < 200 cells/µL versus CD4 ≥ 200 cells/µL groups.

Random-effects models were used to account for the expected variability in effect sizes. This model involves weighting each effect size by its inverse variance, defined as the sum of the within-study variance and between-study variance, the latter being estimated by restricted maximum likelihood [53]. For each meta-analysis, the method proposed by Hartung was applied to compute the mean effect size along with its 95% confidence intervals (CI) [54]. To check the variability in effect sizes, Cochran’s heterogeneity *Q* statistic and the I^2^ index (values of 0%, 25%, 50% and 75% representing no, low, moderate, and high heterogeneity, respectively) were calculated [55]. For each meta-analysis, a forest plot was also constructed.

Publication bias was assessed by constructing funnel plots using the trim-and-fill method, which consists of imputing missing effect sizes to achieve symmetry [56]. Furthermore, Egger’s regression test was also applied [57]. Evidence of publication bias was defined as a statistically significant result for Egger’s test, defined at *p* < 0.10 instead of the usual* p* < 0.05 because of the lower statistical power with a small number of studies [58].

Finally, in the presence of heterogeneity and at least 10 studies for the outcome, analyses of potential effect moderators were performed [59]. Meta-regressions and weighted ANOVAs for continuous and categorical moderators, respectively, were applied by assuming mixed-effect models by means of the *F* statistic, described by Knapp-Hartung [60, 61]. The *Q*^E^ statistic was calculated to assess the model misspecification of the moderator analyses, together with an estimate of the percentage of variance accounted for by the moderator, *R*^2^ [62].

All statistical analyses were carried out with the *metafor* package in *R* version 3.2.3 [63].

## Results

### Characteristics of Included Studies

**Fig. 1:**
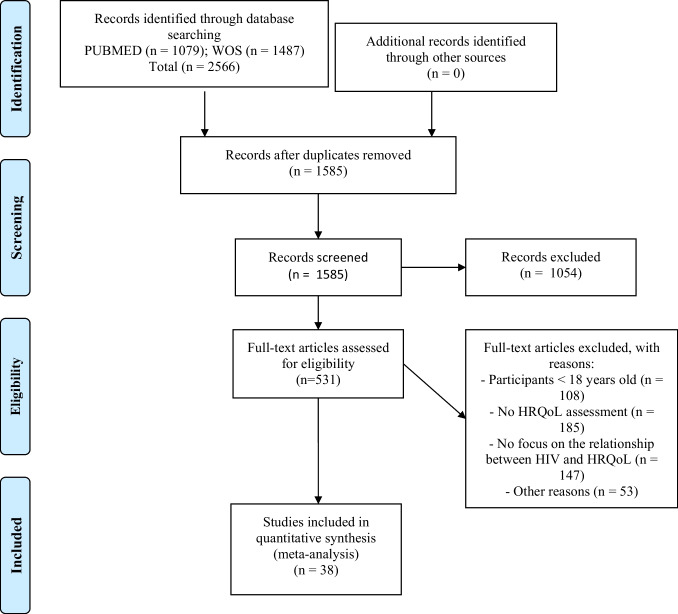
Flow chart of study selection process

The search yielded 1079 results in PubMed and 1487 in the Web of Science. After removing duplicates, a total of 1585 unique records were screened, and 531 full-text articles were assessed for eligibility (see Fig. 1). After exclusion criteria were applied, 38 studies were included in the meta-analysis. One of these used two samples, so a total of 39 samples were analyzed (Table 1; Fig. 1) [24, 27–29, 33, 64–81, 81–95]. According to the assessment with the NOS scale, 18 studies (46.2%) were deemed high quality and 21 (53.8%) moderate quality (Supplementary Table 4). All studies met criteria for the ascertainment of the exposure and the assessment of the outcome, as these were inclusion criteria for the study.

### Mean Effect Sizes and Heterogeneity

**Fig. 2:**
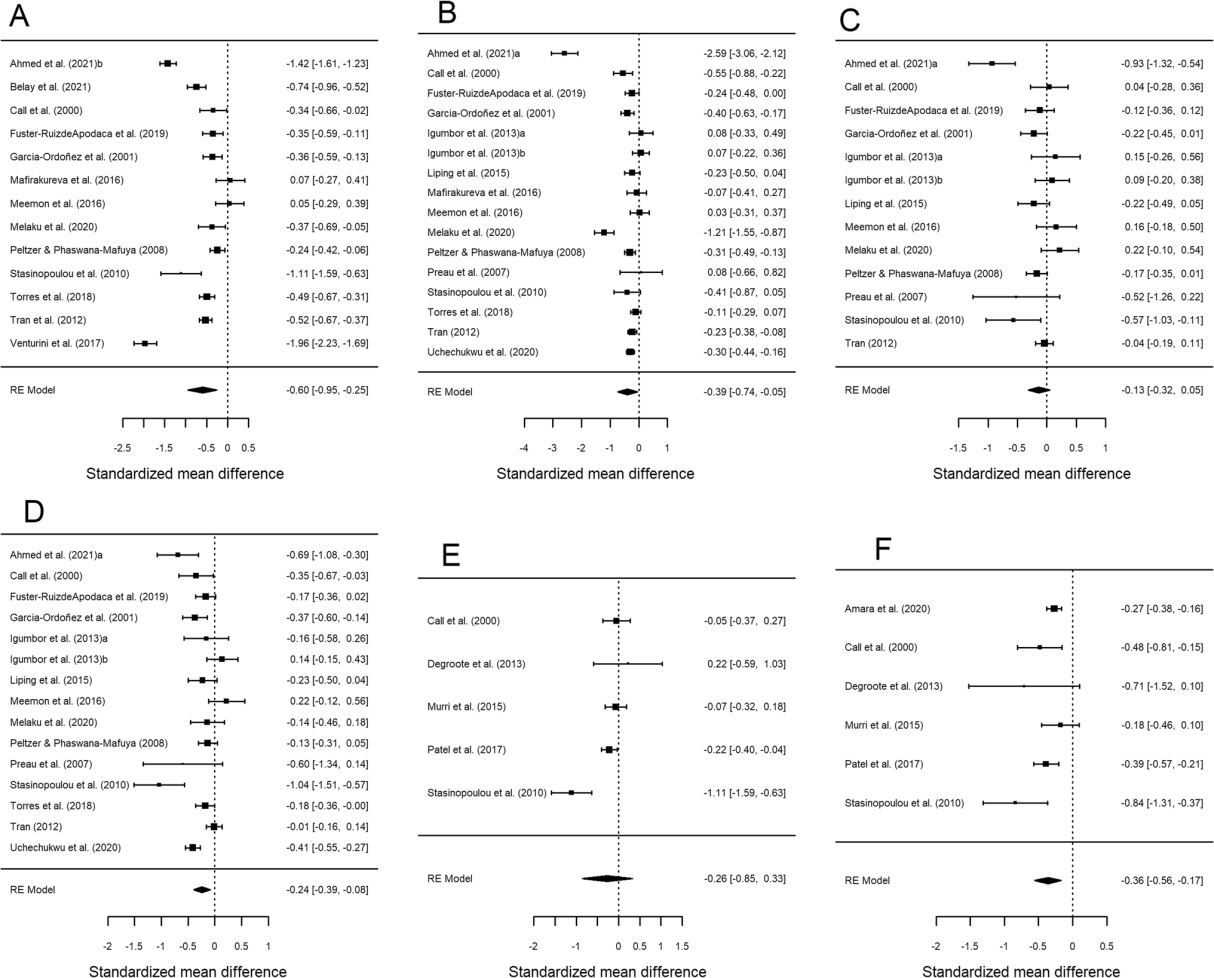
Forest plots displaying the standardized mean differences with 95% confidence intervals for the comparison of the CD4 < 200 cells/μL and CD4 > 200 cells/μL groups for each of the six outcome measures: overall health perception and concern (**A**), physical and functional health and symptoms (**B**), psychological health (**C**), social relationship (**D**), mental health summary (**E**) and physical health summary (**F**). RE (random-effects) model refers to the statistical model used in the calculations

Table 2 presents the results of the meta-analysis for the comparison of the groups with CD4 < 200 cells/µL versus CD4 ≥ 200 cells/µL, for each of the six HRQoL outcome domains. Forest plots are displayed in Fig. 2. The mean effect sizes for all six outcomes were negative, indicating worse HRQoL in the group with lower CD4 counts. All results were statistically significant except for *psychological health* (*d*_+_  − 0.133, 95% CI − 0.319, 0.054) and *mental health summary* (*d*_+_  − 0.260, 95% CI − 0.851, 0.332). The highest mean effect size was found for *overall health perception and concern* (*d*_+_  − 0.598, 95% CI − 0.949, − 0.248), followed by *physical and functional health and symptoms* (*d*_+_  − 0.395, 95% CI − 0.740, − 0.049) and *physical health summary* (*d*_+_  − 0.362, 95% CI − 0.557, − 0.167), with similar magnitudes. The meta-analysis showed heterogeneity (I^2^ > 70% and statistically significant Q), except for *physical health summary*, where the I^2^ value was 36.7% and Cochran’s Q was not significant (Table 2). This large variability is also reflected in the forest plots (Fig. 2).

**Table 2:** Mean effect sizes, 95% confidence intervals, and heterogeneity of the six outcome measures in people with CD4 < 200 cells/µL group versus CD4 ≥ 200 cells/µL

Health-related quality of life domain	*k*	*d*_+_	95% CI	*Q*	I^2^ (%)
Overall health perception and concern	13	− 0.598	− 0.949, − 0.248	233.973^a^	95.7
Physical and functional health and symptoms	16	− 0.395	− 0.740, − 0.049	144.800^a^	95.9
Psychological health	13	− 0.133	− 0.319, 0.054	35.207^a^	74.3
Social relationships	15	− 0.235	− 0.394, − 0.077	49.544^a^	76.2
Mental health summary	5	− 0.260	− 0.851, 0.332	16.922^b^	86.4
Physical health summary	6	− 0.362	− 0.557, − 0.167	8.866	36.7^c^

*k*: number of studies. *d*_+_: mean effect size

*CI* confidence interval, *Q* Cochran’s heterogeneity *Q* statistic; with *k* – 1 degrees of freedom, *I*^2^ heterogeneity index

^a^*p* < 0.001

^b^*p* < 0.01

^c^Due to the lack of heterogeneity found for physical health summary, the mean effect size and the 95% CI was also computed as a sensitivity analysis, applying a fixed-effect model: *d*_+_  =  − 0.323; 95% CI − 0.407, − 0.240

**Fig. 3:**
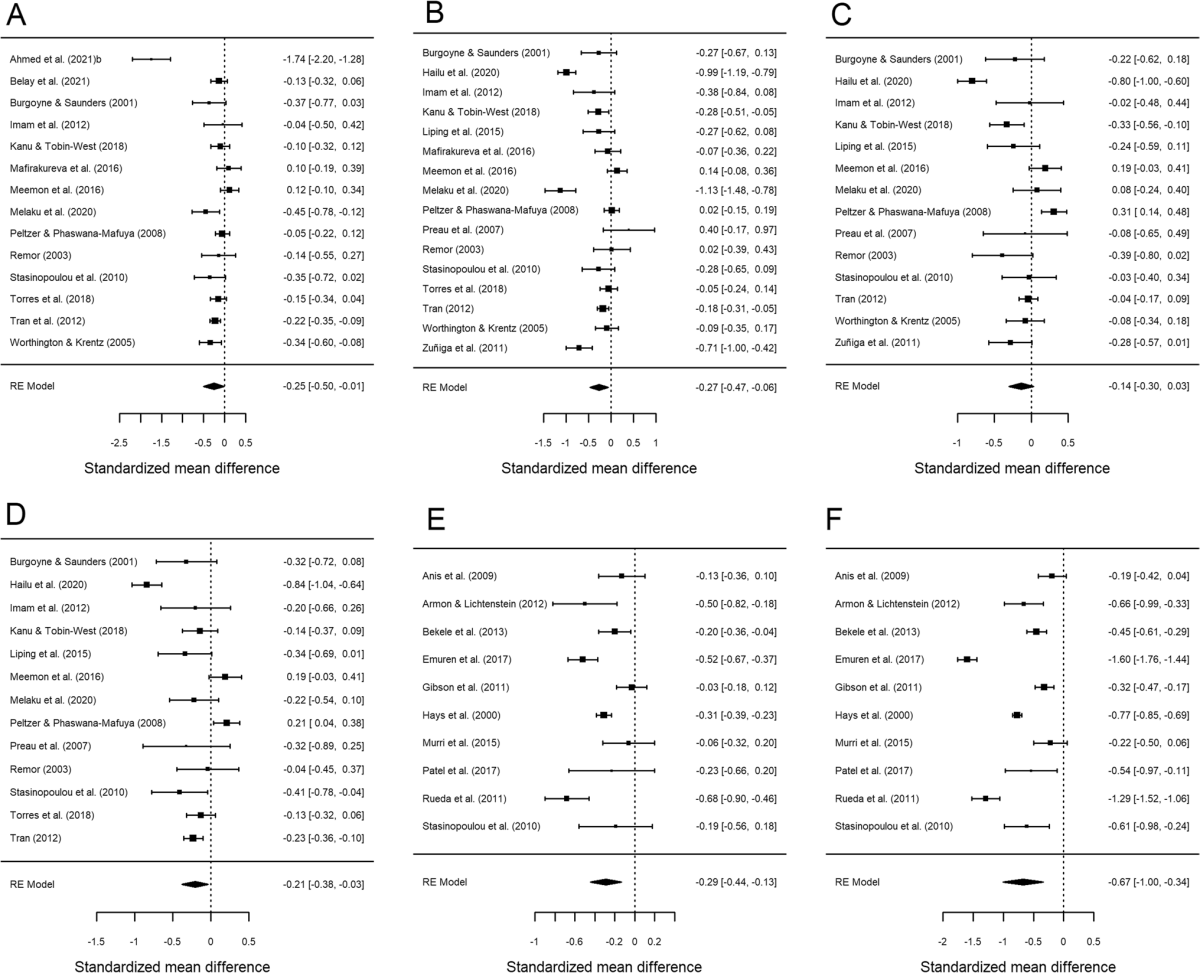
Forest plots displaying the standardized mean differences with 95% confidence intervals for the comparison of the AIDS with non-AIDS groups for each of the six outcome measures: overall health perception and concern (**A**), physical and functional health and symptoms (**B**), psychological health (**C**), social relationship (**D**), mental health summary (**E**) and physical health summary (**F**). RE (random-effects) model refers to the statistical model used in the calculations

Table 3 presents the results of the meta-analysis comparing AIDS versus non-AIDS groups for each of the six HRQoL outcome measures. Forest plots are displayed in Fig. 3. As previously, the mean effect sizes were negative for all six outcomes, indicating poor HRQoL in the AIDS group. All analyses were statistically significant with the exception of *psychological health* (*d*_+_  − 0.136; 95% CI − 0.305, 0.032). In this case, the highest mean effect size was found for *physical health summary* (*d*_+_  − 0.670; 95% CI − 1.002, − 0.338), while the rest of the mean effect sizes showed a low to moderate magnitude. Regarding the variability among effect sizes, very high heterogeneity was found for all outcomes (I^2^ > 80% and statistically significant Q) (Table 3; Fig. 3).

**Table 3:** Mean effect sizes, 95% confidence intervals, and heterogeneity statistics of the six outcome measures for the comparison of the AIDS group versus non-AIDS group

Health-related quality of life domain	k	d_+_	95% CI	Q^a^	I^2^
Overall health perception and concern	14	− 0.254	− 0.499, − 0.009	65.494	90.0%
Physical and functional health and symptoms	16	− 0.265	− 0.475, − 0.056	125.695	88.8%
Psychological health	14	− 0.136	− 0.305, 0.032	88.932	81.4%
Social relationships	13	− 0.206	− 0.378, − 0.033	79.701	81.9%
Mental health summary	10	− 0.287	− 0.444, − 0.131	40.377	81.2%
Physical health summary	10	− 0.670	− 1.002, − 0.338	209.778	95.8%

*k*: number of studies. *d*_+_: mean effect size

*CI* confidence interval, *Q* Cochran’s heterogeneity *Q* statistic; with *k* – 1 degrees of freedom, *I*^2^: heterogeneity index

^a^All analyses yielded *p* < 0.001

### Publication Bias

**Fig. 4:**
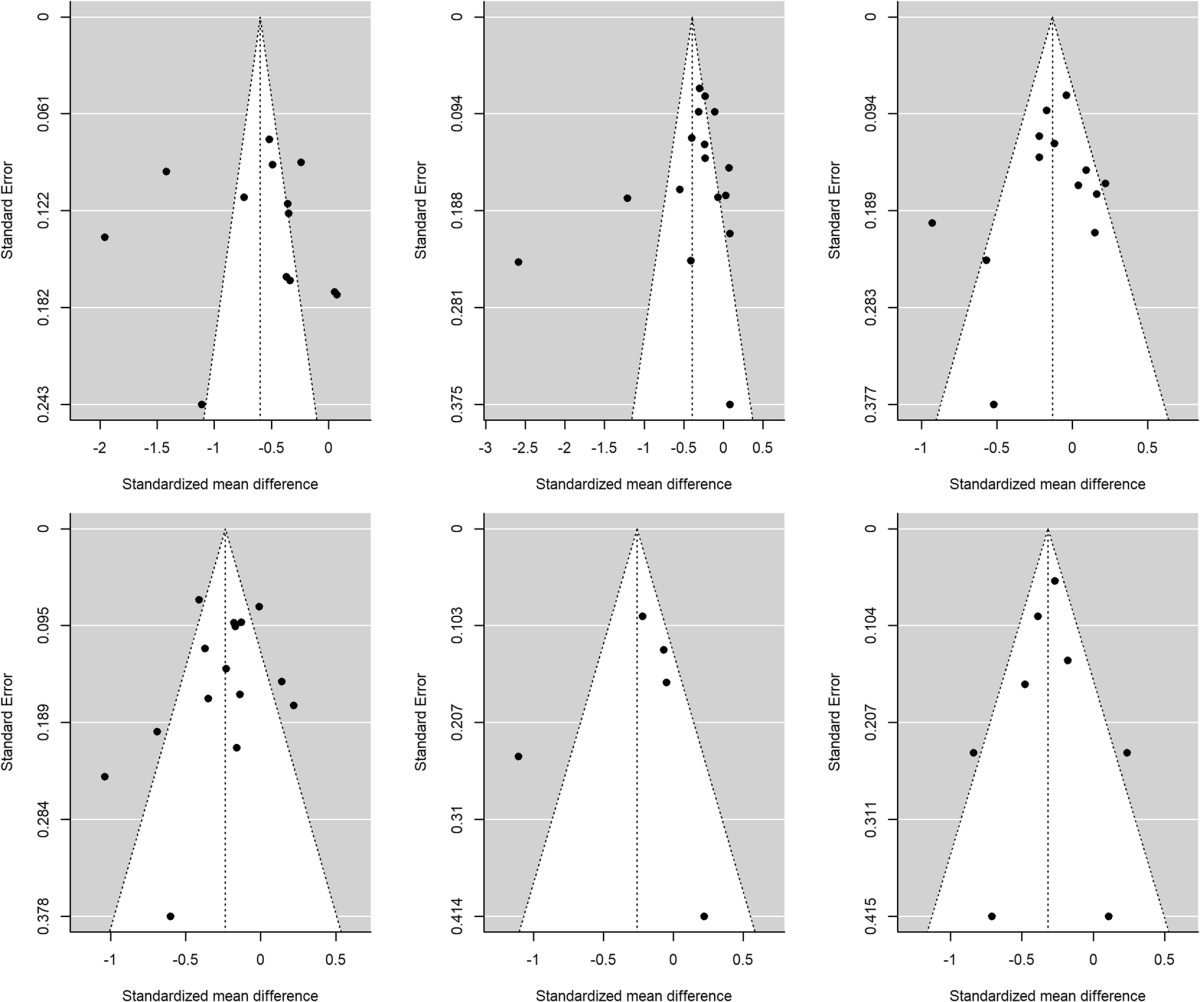
Funnel plots for the comparison of the CD4 < 200 cells/μL and CD4 > 200 cells/μL groups for overall health perception and concern (**A**), physical and functional health and symptoms (**B**), psychological health (**C**), social relationship (**D**), mental health summary (**E**) and physical health summary (**F**) measures. The white circles are the imputed standardized mean changes by means of the Duval and Tweedie's trim and-fill method

Publication bias was assessed through Egger’s tests and funnel plots, using the trim-and-fill method. Figure 4 presents the funnel plots for the comparison according to CD4 counts (< 200 cells/µL versus ≥ 200 cells/µL) for the HRQoL measures. For *physical health summary*, two additional effect sizes were imputed to the original set of estimates to achieve symmetry in the funnel plot (Fig. 4F). When a mean effect (and its 95% CI) was calculated using the six effect sizes plus the two imputed values, the mean effect was *d*_+_  − 0.317 (95% CI − 0.490, − 0.144). However, Egger’s test did not reach statistical significance (*p* = 0.117).

**Fig. 5:**
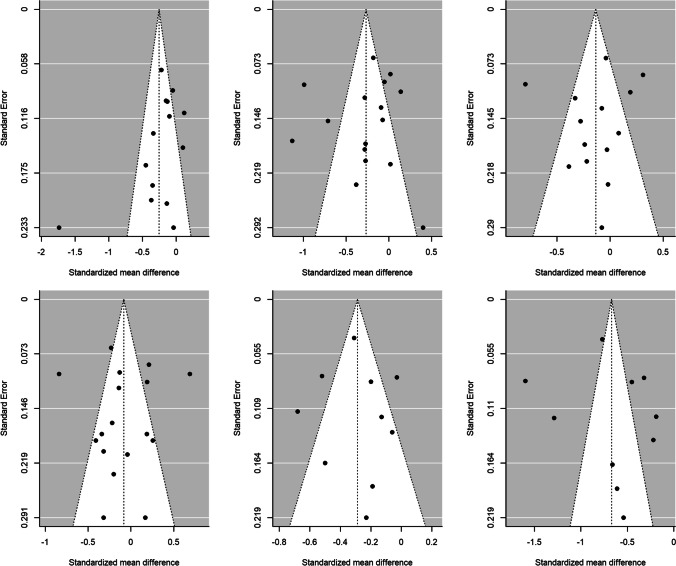
Funnel plots for the comparison of the AIDS with non-AIDS groups for overall health perception and concern (**A**), physical and functional health and symptoms (**B**), psychological health (**C**), social relationship (**D**), mental health summary (**E**) and physical health summary (**F**) measures. The white circles are the imputed standardized mean changes by means of the Duval and Tweedie's trim and-fill method

Figure 5 presents the funnel plots for the comparison of the AIDS versus non-AIDS groups. For *social relationships*, four additional effect sizes were imputed to achieve symmetry in the funnel plot (Fig. 5D). When a mean effect (and its 95% CI) was calculated using the 13 effect sizes plus the four imputed values, the mean effect was *d*_+_  − 0.083 (95% CI − 0.262, 0.095). Nevertheless, once again Egger’s test was not statistically significant (*p* = 0.61).

For the rest of meta-analyses, the trim-and-fill method did not require imputing new values to the funnel plots (Figs. 4, 5), and the Egger’s tests were not statistically significant. Thus, the results of these meta-analyses are at low risk for publication bias.

### Analysis of Moderator Variables

The heterogeneity observed in the standardized mean differences prompted an analysis of moderator variables for outcome measures with at least 10 studies. Tables 4 and 5 show the results of the meta-regressions, and Tables 6 and 7 of the weighted ANOVAs, comparing groups by CD4 counts and AIDS status, respectively.

**Table 4:** Results of the meta-regressions applied to continuous effect modifiers, showing influence on the effect sizes of the outcome measures in participants with CD4 < 200 cells/µL versus CD4 ≥ 200 cells/µL

Effect modifier	*k*	*b*_j_	*F*	*p*	*Q*_E_	*p*	*R*^2^
Overall health perception and concern							
Mean age	11	− 0.077	5.91	**0.038**	94.42	< 0.001	35.5%
Gender (% of men)	13	− 0.010	1.60	0.23	207.88	< 0.001	4.6%
ART (%)	10	− 0.003	0.28	0.61	110.36	< 0.001	0
HIV-VL undetectable (%)	6	− 0.005	0.53	0.51	126.69	< 0.001	0
NOS score	13	0.101	0.19	0.67	229.77	< 0.001	0
Physical and functional health and symptoms							
Mean age	14	0.002	0.01	0.94	49.53	< 0.001	0
% men	16	− 0.008	0.89	0.36	140.29	< 0.001	0
% ART	14	− 0.005	1.02	0.33	139.45	< 0.001	0
% undetectable HIV-VL	5	− 0.006	0.15	0.73	93.65	< 0.001	0
NOS score	16	− 0.275	1.38	0.26	142.98	< 0.001	2.0%
Psychological health							
Mean age	11	0.005	0.08	0.79	16.43	0.058	0
% men	13	− 0.006	2.44	0.15	32.49	< 0.001	8.3%
% ART	11	− 0.003	1.07	0.33	28.74	< 0.001	0
% undetectable HIV-VL	4	− 0.004	0.35	0.62	15.10	< 0.001	0
NOS score	13	0.119	0.76	0.40	31.53	< 0.001	0
Social relationships							
Mean age	13	0.007	0.07	0.80	41.39	< 0.001	0
% men	15	− 0.007	5.08	**0.042**	40.11	< 0.001	30.8%
% ART	13	− 0.002	0.74	0.41	32.12	< 0.001	0
% undetectable HIV-VL	5	− 0.001	0.08	0.80	7.44	0.059	0
NOS score	15	0.057	0.27	0.61	49.37	< 0.001	0

In bold, results for effect modifiers that reached statistical significance (*p* < 0.05)

*ART* antiretroviral treatment, *NOS* Newcastle–Ottawa scale, *VL* viral load

*k*—number of studies, *b*_j_—regression coefficient, *F*—*F* statistic to test the statistical significance of the moderator, *Q*_E_—statistic for testing the model misspecification, *R*^2^—percentage of variance accounted for by the moderator

**Table 5:** Results of the meta-regressions applied to continuous effect modifiers, showing influence on the effect sizes of the outcome measures in participants with versus without AIDS

Effect modifier	*k*	*b*_j_	*F*	*p*	*Q*_E_	*p*	*R*^2^
Overall health perception and concern							
Mean age	12	− 0.001	0.00	0.98	17.60	0.062	0
% men	14	− 0.007	1.89	0.19	53.11	< 0.001	11.7%
% ART	12	− 0.002	0.07	0.78	63.92	< 0.001	0
% undetectable HIV-VL	4	− 0.003	0.05	0.85	43.59	< 0.001	0
NOS score	14	− 0.146	1.13	0.31	63.62	< 0.001	0
Physical and functional health and symptoms							
Mean age	15	0.032	0.55	0.47	110.09	< 0.001	0
% men	16	0.001	0.03	0.87	125.48	< 0.001	0
% ART	13	− 0.005	0.54	0.48	103.38	< 0.001	0
% undetectable HIV-VL	3	0.007	0.70	0.56	2.81	0.094	0
NOS score	16	− 0.130	1.09	0.32	122.52	< 0.001	0
Psychological health							
Mean age	13	0.048	3.29	0.097	55.48	< 0.001	16.3%
% men	14	− 0.003	0.44	0.52	85.28	< 0.001	0
% ART	11	− 0.006	1.34	0.28	61.47	< 0.001	6.6%
% undetectable HIV-VL	14	− 0.039	0.13	0.73	83.16	< 0.001	0
Social relationships							
Mean age	12	0.020	0.41	0.54	50.75	< 0.001	0
% men	13	− 0.005	1.45	0.25	70.22	< 0.001	2.7%
% ART	11	− 0.004	0.59	0.46	61.95	< 0.001	0
% undetectable HIV-VL	3	− 0.004	3.67	0.31	0.21	0.649	0
NOS score	13	− 0.002	0.00	0.98	77.65	< 0.001	0
Mental health summary							
Mean age	9	0.016	0.51	0.50	32.97	< 0.001	0
% men	10	− 0.006	1.54	0.25	33.24	< 0.001	12.6%
% ART	6	− 0.001	0.16	0.71	14.73	0.005	0
% undetectable HIV-VL	5	− 0.002	0.04	0.85	32.87	< 0.001	0
NOS score	10	0.152	2.44	0.16	28.61	< 0.001	20.3%
Physical health summary							
Mean age	9	0.041	0.79	0.41	165.67	< 0.001	0
% men	10	− 0.008	0.89	0.37	178.21	< 0.001	0.07%
% ART	6	− 0.002	0.09	0.77	140.75	< 0.001	0
% undetectable HIV-VL	5	0.003	0.03	0.87	133.62	< 0.001	0
NOS score	10	0.331	2.65	0.14	151.58	< 0.001	16.3%

*ART* antiretroviral treatment, *NOS* Newcastle–Ottawa scale, *VL* viral load

*k*—number of studies, *b*_j_—regression coefficient, *F*—*F* statistic to test the statistical significance of the moderator, *Q*_E_—statistic for testing the model misspecification, *R*^2^—percentage of variance accounted for by the moderator

**Table 6:** Results of the ANOVAs for the influence of categorical effect modifiers on the outcome measures in participants with CD4 < 200 cells/µL versus CD4 ≥ 200 cells/µL

Effect modifier	*k*	*d*_+_	95% CI	ANOVA results
Overall health perception and concern				
Country income level, according to World Bank classification:				
Low income	2	− 0.558	− 1.705, 0.590	*F*_3,8_ = 0.05, *p* = .99 *R*^2^ = 0 *Q*_E_(8) = 217.55, *p* < .001
Lower-middle income	3	− 0.633	− 1.565, 0.300	
Upper-middle income	3	− 0.719	− 1.655, 0.218	
High income	4	− 0.529	− 1.348, 0.289	
Recruitment period:				*F*_2,9_ = 0.33, *p* = .73 *R*^2^ = 0 *Q*_E_(9) = 199.52, *p* < .001
1996–2003	2	− 0.350	− 1.353, 0.652	
2004–2011	1	− 0.240	− 1.636, 1.156	
2012–2019	9	− 0.643	− 1.113, − 0.172	
QOL questionnaire:				*F*_1,11_ = 1.01, *p* = .34 *R*^2^ = 1.14% *Q*_E_(1) = 196.56, *p* < .001
General	8	− 0.724	− 1.173, − 0.276	
Specific	5	− 0.390	− 0.967, 0.187	
Physical and functional health and symptoms				
Country income level, according to World Bank classification:				*F*_3,11_ = 1.27, *p* = .33 *R*^2^ = 3.82% *Q*_E_(11) = 128.49, *p* < .001
Low income	2	− 0.740	− 1.732, 0.252	
Lower-middle income	3	− 0.916	− 1.738, − 0.093	
Upper-middle income	5	− 0.076	− 0.709, 0.557	
High income	5	− 0. 320	− 0.970, 0.329	
Recruitment period:				*F*_2,10_ = 0.22, *p* = .81 *R*^2^ = 0 *Q*_E_(10) = 132.33, *p* < .001
1996–2003	2	− 0.474	− 1.646, 0.698	
2004–2011	2	− 0.137	− 1.359, 1.085	
2012–2019	9	− 0.533	− 1.086, 0.020	
QOL questionnaire:				*F*_1,14_ = 0.16, *p* = .70 *R*^2^ = 0 *Q*_E_(14) = 143.81, *p* < .001
General	4	− 0.282	− 0.989, 0.426	
Specific	12	− 0.434	− 0.849, − 0.019	
Psychological health				
Country income level, according to World Bank classification:				*F*_3,9_ = 1.49, *p* = .28 *R*^2^ = 0 *Q*_E_(9) = 29.28, *p* < .001
Low income	1	0.220	− 0.437, 0.877	
Lower-middle income	2	− 0.391	− 0.837, 0.054	
Upper-middle income	5	− 0.016	− 0.304, 0.272	
High income	5	− 0.223	− 0.533, 0.087	
Recruitment period:				*F*_2,7_ = 0.13, *p* = .88 *R*^2^ = 0 *Q*_E_(7) = 27.14, *p* < .001
1996–2003	2	− 0.098	− 0.671, 0.475	
2004–2011	2	− 0.273	− 0.917, 0.370	
2012–2019	6	− 0.139	− 0.473, 0.194	
QOL questionnaire:				*F*_1,11_ = 0.03, *p* = .86 *R*^2^ = 0 *Q*_E_(11) = 35.13, *p* < .001
General	2	− 0.100	− 0.584, 0.385	
Specific	11	− 0.142	− 0.360, 0.075	
Social relationships				
Country income level, according to World Bank classification:				*F*_3,10_ = 1.62, *p* = .25 *R*^2^ = 4.51% *Q*_E_(10) = 31.47, *p* < .001
Low income	2	− 0.296	− 0.710, 0.118	
Lower-middle income	2	− 0.275	− 0.704, 0.154	
Upper-middle income	5	− 0.037	− 0.316, 0.242	
High income	5	− 0.434	− 0.733, − 0.136	
Recruitment period:				*F*_2,9_ = 0.42, *p* = .67 *R*^2^ = 0 *Q*_E_(9) = 29.25, *p* < .001
1996–2003	2	− 0.361	− 0.733, 0.010	
2004–2011	2	− 0.212	− 0.629, 0.205	
2012–2019	8	− 0.195	− 0.371, − 0.018	
QOL questionnaire				*F*_1,13_ = 0.15, *p* = .70 *R*^2^ = 0 *Q*_E_(13) = 48.65, *p* < .001
General	3	− 0.293	− 0.647, 0.060	
Specific	12	− 0.221	− 0.410, − 0.033	

*k*—number of studies, *d*_+_—mean effect size, CI—confidence interval, *F*—*F* statistic for testing the statistical significance of the effect modifier, *R*^2^—percentage of variance accounted for by the modifier, *Q*_E_—statistic for testing the model misspecification

**Table 7:** Results of the ANOVAs for the influence of categorical effect modifiers on the outcome measures in participants with versus without AIDS

Effect modifier	*k*	*d*_+_	95% CI	ANOVA results
Overall health perception and concern				
Country income level, according to World Bank classification:				*F*_3,9_ = 0.46, *p* = .717 *R*^2^ = 0 *Q*_E_(9) = 53.29, *p* < .001
Low income	3	− 0.221	− 0.840, 0.398	
Lower-middle income	4	− 0.440	− 0.995, 0.115	
Upper-middle income	2	0.034	− 0.711, 0.779	
High income	4	− 0.302	− 0.859, 0.256	
Recruitment period:				*F*_2,9_ = 0.12, *p* = .885 *R*^2^ = 0 *Q*_E_(9) = 63.38, *p* < .001
1996–2003	2	− 0.255	− 1.103, 0.593	
2004–2011	3	− 0.147	− 0.816, 0.522	
2012–2019	7	− 0.322	− 0.754, 0.111	
QOL questionnaire:				*F*_1,12_ = 0.56, *p* = .468 *R*^2^ = 0 *Q*_E_(12) = 63.08, *p* < .001
General	7	− 0.342	− 0.700, 0.016	
Specific	7	− 0.169	− 0.524, 0.188	
Physical and functional health and symptoms				
Country income level, according to World Bank classification:				*F*_3,11_ = 3.35, *p* = .059 *R*^2^ = 38.21% *Q*_E_(11) = 56.384, *p* < .001
Low income	3	− 0.786	− 1.186, − 0.385	
Lower-middle income	3	− 0.194	− 0.606, 0.217	
Upper-middle income	4	− 0.186	− 0.533, 0.161	
High income	5	− 0.072	− 0.416, 0.272	
Recruitment period:				*F*_2,11_ = 0.79, *p* = .479 *R*^2^ = 0 *Q*_E_(11) = 110.91, *p* < .001
1996–2003	3	− 0.334	− 0.893, 0.225	
2004–2011	4	− 0.026	− 0.512, 0.461	
2012–2019	7	− 0.356	− 0.704, − 0.007	
QOL questionnaire:				*F*_1,14_ = 1.05, *p* = .323 *R*^2^ = 1.07% *Q*_E_(14) = 118.74, *p* < .001
General	4	− 0.090	− 0.513, 0.333	
Specific	12	− 0.323	− 0.565, − 0.081	
Psychological health				
Country income level, according to World Bank classification:				*F*_3,10_ = 1.41, *p* = .297 *R*^2^ = 15.19% *Q*_E_(10) = 42.07, *p* < .001
Low income	3	− 0.381	− 0.724, − 0.038	
Lower-middle income	2	− 0.033	− 0.469, 0.403	
Upper-middle income	4	0.023	− 0.276, 0.322	
High income	5	− 0.157	− 0.461, 0.146	
Recruitment period:				*F*_2,9_ = 1.17, *p* = .353 *R*^2^ = 2.97% *Q*_E_(9) = 65.09, *p* < .001
1996–2003	3	− 0.295	− 0.708, 0.118	
2004–2011	4	0.059	− 0.297, 0.415	
2012–2019	5	− 0.167	− 0.460, 0.126	
QOL questionnaire:				*F*_1,12_ = 0.63, *p* = .443 *R*^2^ = 0 *Q*_E_(12) = 87.01, *p* < .001
General	2	− 0.304	− 0.796, 0.188	
Specific	12	− 0.113	− 0.296, 0.071	
Social relationships				
Country income level, according to World Bank classification:				*F*_3,8_ = 1.71, *p* = .241 *R*^2^ = 22.52% *Q*_E_(8) = 33.64, *p* < .001
Low income	3	− 0.418	− 0.765, − 0.072	
Lower-middle income	2	− 0.219	− 0.660, 0.221	
Upper-middle income	3	0.052	− 0.294, 0.398	
High income	4	− 0.272	− 0.630, 0.086	
Recruitment period:				*F*_2,8_ = 0.43, *p* = .665 *R*^2^ = 0 *Q*_E_(8) = 58.98, *p* < .001
1996–2003	2	− 0.181	− 0.743, 0.381	
2004–2011	3	− 0.045	− 0.502, 0.411	
2012–2019	6	− 0.262	− 0.550, 0.027	
QOL questionnaire:				*F*_1,11_ = 0.09, *p* = .770 *R*^2^ = 0 *Q*_E_(11) = 79.44, *p* < .001
General	3	− 0.160	− 0.543, 0.223	
Specific	10	− 0.219	− 0.424, − 0.015	
Mental health summary				
Country income level, according to World Bank classification:				*F*_1,8_ = 0.04, *p* = .845 *R*^2^ = 0 *Q*_E_(8) = 40.29, *p* < .001
Lower-middle income	1	− 0.230	− 0.908, 0.448	
High income	9	− 0.291	− 0.466, − 0.117	
Recruitment period:				*F*_1,5_ = 0.16, *p* = .704 *R*^2^ = 0 *Q*_E_(5) = 26.70, *p* < .001
1996–2003	2	− 0.206	− 0.581, 0.169	
2004–2011	5	− 0.277	− 0.534, − 0.020	
QOL questionnaire:				*F*_1,7_ = 0.09, *p* = .765 *R*^2^ = 0 *Q*_E_(7) = 38.23, *p* < .001
General	4	− 0.279	− 0.552, − 0.006	
Specific	5	− 0.328	− 0.588, − 0.069	
Physical health summary				
Country income level, according to World Bank classification:				*F*_1,8_ = 0.07, *p* = .800 *R*^2^ = 0 *Q*_E_(8) = 208.93, *p* < .001
Lower-middle income	1	− 0.540	− 1.738, 0.658	
High income	9	− 0.682	− 1.057, − 0.308	
Recruitment period:				*F*_1,5_ = 0.14, *p* = .727 *R*^2^ = 0 *Q*_E_(5) = 175.29, *p* < .001
1996–2003	2	− 0.504	− 1.464, 0.456	
2004–2011	5	− 0.668	− 1.284, − 0.051	
QOL questionnaire:				*F*_1,7_ = 0.28, *p* = .613 *R*^2^ = 0 *Q*_E_(7) = 178.19, *p* < .001
General	4	− 0.818	− 1.381, − 0.254	
Specific	5	− 0.647	− 1.158, − 0.136	

*k*—number of studies, *d*_+_—mean effect size, CI—confidence interval, *F*—*F* statistic for testing the statistical significance of the effect modifier, *R*^2^—percentage of variance accounted for by the modifier, *Q*_E_—statistic for testing the model misspecification

Of the different potential moderator variables analyzed, only two continuous variables yielded a statistically significant result for the comparison of the CD4 groups (see Table 4): mean age exhibited a statistically significant relationship with the effect size for *overall health perception and concern* (*p* = 0.038), accounting for 35.5% of the variance observed. The negative sign of the regression coefficient for this moderator indicated larger standardized mean differences, as the mean age of participants decreased for this outcome measure. On the other hand, the percentage of men showed a significant relationship with the effect sizes of the *social relationships* measure (*p* = 0.042), accounting for 30.8% of the variance. Once again, a negative relationship was found, such that the larger the proportion of men, the lower the standardized mean differences for this outcome measure.

Finally, for the comparison of groups with AIDS versus without AIDS, country income level yielded a marginally significant result (*p* = 0.059) as an effect modifier, explaining a notable percentage of the variance (*R*^2^ = 38.21%) for the *physical and functional health and symptoms* measure (see Table 7). The highest effect was found in the low-income category (*d*_+_  − 0.786).

## Discussion

We conducted a meta-analysis to assess the influence of AHD on HRQoL and its different domains. We included papers published over the last 25 years of the AIDS pandemic, analyzing possible differences in HRQoL and AHD in different periods of time as well as differences between countries of different income levels. Our main finding was that HRQoL is worse in patients with AHD compared to those without. People with CD4 counts of fewer than 200 cells/µL sustain negative impacts in all domains of HRQoL, especially in *overall health perception and concern*, followed by *physical and functional health and symptoms*, and *physical health summary*, with similar mean effect sizes but large inter-study variability. The outcomes related to *psychological health* and *mental health summary* were also negatively affected, though comparisons did not reach statistical significance. In patients with an AIDS diagnosis, the six HRQoL domains showed lower scores than in those without AIDS. The greatest difference was observed for *physical health summary*. As in patients with low CD4 cells, *psychological health* scores did not show a significant difference between people with versus without AIDS. This finding is probably related to the negative psychological impact of an HIV diagnosis on mental health in PLHIV, regadless of AIDS diagnosis or number of CD4 cells [96, 97]. PLHIV often experience intersecting types of discrimination (marginalized identities, internalized HIV stigma, limited economic resources, etc.) or suffer from uncertainty about a non-curable and potentially lethal infection. Thus, PLHIV are highly vulnerable to mental health problems [98, 99], independently of their immunovirological status.

In this meta-analysis, we found that *overall health perception and concern* and *physical and functional health and symptoms* are the main HRQoL domains affected in PLHIV with AHD. *Physical and functional health and symptoms* includes different dimensions, including physical functioning, energy, mobility, effects and severity of pain, and level of independence. The symptomatology of advanced stages of HIV infection, the associated comorbidities, and the loss of vitality caused by progression of HIV infection could explain the worse scores in these domains [19–22].

Our meta-analysis considers numerous effect modifiers (age, gender, treatment with ART, and HIV viral load) that were analyzed for their potential influence on the effect sizes of the HRQoL outcome. We found a moderate impact for age and gender on some HRQoL domains. Age showed an inverse relationship with the effect sizes for *overall health perception and concern*, such that older age was associated with worse scores, probably due to ageing, comorbidities, and disability associated with AHD [100]. The domain for *overall health perception and concern* comprises perceptions, distress, concerns, and worries related to general health. Thus, this result is consistent with previous studies showing that HRQoL indexes of physical health are negatively affected in older PLHIV [101]. Furthermore, male sex showed a significant negative relationship with the effect sizes for *social relationships*. In high-income countries, men with HIV are usually reported as having better scores in *social relationships* and other related dimensions or predictors than women with HIV [27, 31]. Our results shows that this trend could differ between countries depending on income level, that AHD could affect men’s social relationships more than women’s, or that in low- and medium-income countries women use social support as coping strategy to reduce the stressors on health outcomes [8, 102]. Another possibility is that men are overrepresented in this meta-analysis, especially in studies from low- and middle-income countries.

Regarding the relationship between HRQoL and country income level, we hypothesized that people with AHD from middle- or high-income countries would report higher HRQoL scores than in those living in low-income countries, due to the direct impact of socioeconomic status on HRQoL [8]. However, we did not find differences between the analyzed countries according to this parameter. When we compared the group with versus without AIDS, we found only a borderline significant result for *physical and functional health and symptoms*, in PLHIV with AIDS from low-income countries. These results could be related with worse conditions in housing, nutrition, health resources, employment difficulties, and other socioeconomic indicators in these settings [88, 95]. In fact, there is evidence supporting the degenerative impact that material deprivation has on HRQoL [103].

Furthermore, given the continuous improvements in the efficacy and safety of ART from 1996 to the present, we hypothesized that HRQoL in PLHIV with AHD in the era of the new ART (2012–2019) would be better than when these drugs were first introduced (1996). We analyzed three 8-year periods: 1996–2003, 2004–2011, and 2012–2019. Surprisingly, we observed no differences between the time periods analyzed. AHD continues being the worst condition in the spectrum of HIV infection, occurring in high-income and low-income countries alike, despite the availability of new antiretroviral treatments, early diagnosis strategies, and easy access to ART. Ghiasvand et al.’s meta-analysis of studies from 2005 to 2016 in low-, middle- and high-income countries likewise found no impact on HRQoL from ART in PLHIV [103]. The present meta-analysis broadens this evidence by also including earlier periods with less effective and more toxic treatments in PLHIV with AHD. These results suggest that improving quality of life for PLHIV may require additional interventions beyond just the provision of ART.

To address the significant impact of AHD on HRQoL, the initial step should be the implementation of prevention programs, early diagnosis, and early treatment to decrease the prevalence of AHD worldwide [44]. Regarding strategies to enhance HRQoL in PLHIV, these should first focus on addressing basic needs such as nutrition, access to healthcare resources, and employment, which have been associated with low HRQoL [6, 95]. This is particularly crucial in low-income countries, where inequalities are often more pronounced and require steadfast policy responses. Indeed, a systemic and comprehensive approach that considers the special individual needs of this population is essential everywhere. This should include bolstering resilience resources, economic empowerment, and self-stigma detection. Moreover, PLHIV with AHD need preventive interventions that focus on both AIDS and non-AIDS events, among other aspects [6]. Other strategies should provide health education along with comprehensive disease management information and training to ensure adherence to ART, thereby empowering patients to effectively participate in controlling their disease [76]. Emotional support services and counseling should be offered to manage stress and other psychological challenges [29]. Encouraging participation in support groups, social activities, and community programs is also crucial for reducing loneliness [74]. Additionally, efforts should be directed towards eradicating the stigma associated with AHD while promoting inclusivity and understanding for PLHIV in society [25]. To achieve all these goals, individuals with AHD should receive multidisciplinary, comprehensive, and personalized care from a team of physicians, nurses, social workers, and psychologists.

Our study has some limitations. First of all, we could only make use of data available from published studies. Second, we were unable to explore the influence of some factors associated with reduced HRQoL: intravenous drug use or substance misuse, socioeconomic inequalities, refugee or migrant status, lower educational level, social support, and internalized stigma [104]. Third, some countries and WHO regions are underrepresented. We did not find data from Latin America (except from Mexico and Guyana), eastern Europe countries, the Western Pacific and Eastern Mediterranean regions (except Pakistan), India, Brazil, or others. The languages used in this meta-analysis (Spanish or English), along with the databases chosen (PubMed and the Web of Science, both dominated by research from Western countries) probably generated some selection bias in the included studies.

On the other hand, the review also has some important strengths. First, we obtained enough data to demonstrate the highly negative impact of AHD on HRQoL. Second, the long study period enabled a comparison of HRQoL in different periods of the AIDS pandemic and in countries with different income levels. Its results show that despite all the advances in the treatment of HIV infection over the last 25 years, AHD persists as a source of extreme vulnerability for PLHIV.

In conclusion, this meta-analysis shows that AHD has a negative impact on the health and well-being of PLHIV, affecting all HRQoL domains, especially overall self-perceived health, physical health summary, and psychological health. These effects have not changed in the last 25 years and affect all PLHIV with AHD independently of country of residence. HIV clinicians and researchers should focus future studies on improving HRQoL and better understanding the special needs of this vulnerable population.

### Supplementary Information

Below is the link to the electronic supplementary material.

Supplementary file1 (DOCX 33 KB)

Supplementary file1 (DOCX 32 KB)

## Data Availability

Dataset is available upon request.
