# Supplementary material for: Health-Related Quality of Life in People with Advanced HIV Disease, from 1996 to 2021: Systematic Review and Meta-analysis
Source: AIDS Behav. 2024 May 14;28(6):1978–98. doi: 10.1007/s10461-024-04298-y (PMC11161547; doi:10.1007/s10461-024-04298-y)
Supplement: Supplementary file 2 — Supplementary file1 (DOCX 32 KB) [file 10461_2024_4298_MOESM2_ESM.docx]

| **Supplementary Table 2. Full Search Strategy in Pubmed and Web of Sciences (WOS)** | | |
| --- | --- | --- |
| **Pubmed** |  |  |
| **#1** | “HIV”[Mesh] OR “Adquired Immunodeficiency Syndrome”[Mesh] OR “advanced HIV disease” [Title/Abstract] OR “low CD4 cells” [Title/Abstract] | |
| **#2** | “Quality of life”[Mesh] OR “health-related quality of life”[Title/Abstract] OR “HRQOL”[Title/Abstract] | |
| **#3** | #1 AND #2 | |
|  |  | |
| **Web of Sciences (WOS)** | | |
| **#1** | TS = (HIV OR AIDS OR “advanced HIV disease” OR “low CD4 cells”) | |
| **#2** | TS= (“Quality of life” OR “health-related quality of life” OR “HRQOL”) | |
| **#3** | #1 AND #2 | |

| **Supplementary Table 3.** Description of how dimensions of the questionnaires were categorized into domains for the present study | | |
| --- | --- | --- |
| **QoL domains** | **Dimensions from questionnaires** | **HRQoL Questionnaire** |
| **Overall general health perception** | Overall QoL | WHOQOL-HIV-BRIEF |
|  | Overall general health perception | WHOQOL-HIV-BRIEF |
|  | Quality of life | MOS-HIV/MOS-SF-30 |
|  | General health perceptions | MOS-SF-30/ MOS-SF-36/ |
|  | Overall health | EQ-5D-5L/ EQ-5D-3L/ PROQoL-HIV |
|  | Health distress | MOS-HIV/MOS-SF-30 |
|  | Health concern | PROQoL-HIV |
|  | Health worries | HAT–QoL |
| **Physical and**  **functional**  **health and**  **symptoms** | Physical health | WHOQOL-HIV-BRIEF/MOS-HIV/MOS-SF-36 |
|  | Physical health and symptom | PROQoL-HIV |
|  | Physical function | PROMIS-29 |
|  | Overall function | HAT–QoL |
|  | Level of independence | WHOQOL-HIV-BRIEF |
|  | Role functioning | MOS-HIV/MOS-SF-30 |
|  | Energy/fatigue | MOS-HIV |
|  | Vitality/energy/fatigue | MOS-SF-36/MOS- SF30 |
|  | Fatigue | PROMIS-29 |
|  | Pain | MOS-HIV |
|  | Bodily pain | MOS-SF-36/MOS-SF30 |
|  | Pain interference | PROMIS-29 |
|  | Pain intensity | PROMIS-29 |
|  | Role-physical | MOS-HIV |
|  | Cognitive functioning | MOS-HIV |
| **Psychological**  **health** | Psychological health | WHOQOL-HIV-BRIEF |
|  | Mental health | MOS-HIV |
|  | Mental health | MOS-SF-36/MOS-SF-30/ |
|  | Emotional well-being | MOS-SF-36/MOS-SF-30 |
|  | Spirituality | WHOQOL-HIV-BRIEF |
|  | Role-emotional | MOS-HIV/MOS-SF-30 |
|  | Emotional distress | PROQoL-HIV |
| **Social**  **relationships** | Social relations | WHOQOL-HIV-BRIEF |
|  | Social functioning | MOS-HIV/ MOS-SF-36/MOS-SF-30 |
|  | Social relationship | PROQoL-HIV |
|  | Intimate relationships | PROQoL-HIV |
|  | Ability to participate in social roles and activities | PROMIS 29 |
| **Mental health**  **Summary** | Mental health summary | MOS-HIV/MOS-SF-36 |
| **Physical health**  **Summary** | Physical health summary | MOS-HIV/MOS-SF-36 |

|  | | | | | | | | |
| --- | --- | --- | --- | --- | --- | --- | --- | --- |
| **Supplementary Table 4.** Newcastle-Ottawa scale (NOS). Quality assessment scale of individual studies (adapted for cross- sectional studies) | | | | | | | | |
| **Author (Year)** | **Sample representativeness** | **Sample size** | **Non-respondents** | **Ascertainment of the exposure** | **Comparability** | **Assessment of the outcome** | **Statistical test** | **NOS score** |
| Ahmed et al. 2021a | * | * | - | ** | ** | * | * | 8 |
| Ahmed et al. 2021b | ** | * | - | ** | ** | * | * | 9 |
| Amara et al. 2020 | * | - | - | ** | ** | * | * | 7 |
| Anis et al. 2009 | ** | * | - | ** | ** | * | * | 9 |
| Armon & Lichtenstein 2012 | * | - | - | ** | ** | * | * | 7 |
| Bekele et al. 2013 | * | - | - | ** | ** | * | * | 7 |
| Belay et al. 2011 | * | * | - | ** | ** | * | * | 8 |
| Burgoyne & Saunders 2001 | * | - | - | ** | ** | * | * | 7 |
| Call et al. 2000 | ** | - | - | ** | ** | * | * | 8 |
| Degroote et al. 2013 | * | * | - | ** | ** | * | * | 8 |
| Emuren et al. 2017 | * | - | - | ** | ** | * | * | 7 |
| Fuster-RuizdeApodaca et al. 2019 | * | * | - | ** | ** | * | * | 8 |
| Garcia-Ordoñez et al. 2001 | * | - | - | ** | ** | * | * | 7 |
| Gibson et al. 2011 | * | - | * | ** | ** | * | * | 8 |
| Hailu et al. 2020 | * | * | - | ** | ** | * | * | 8 |
| Hays et al. 2000 | * | - | - | ** | ** | * | * | 7 |
| Igumbor et al. 2013a | * | - | - | ** | ** | * | * | 7 |
| Igumbor et al. 2013b | * | - | - | ** | ** | * | * | 7 |
| Imam et al. 2012 | * | * | - | ** | ** | * | * | 7 |
| Kanu & Tobin-West 2018 | ** | * | - | ** | ** | * | * | 9 |
| Liping et al. 2015 | * | - | - | ** | ** | * | * | 7 |
| Mafirakureva et al. 2016 | * | * | - | ** | ** | * | * | 8 |
| Meemon et al. 2016 | * | * | - | ** | ** | * | * | 8 |
| Melaku et al. 2020 | ** | * | - | ** | ** | * | * | 9 |
| Murri et al. 2015 | * | - | - | ** | ** | * | * | 7 |
| Patel et al. 2017 | ** | - | - | ** | ** | * | * | 8 |
| Peltzer & Phaswana-Mafuya, 2008 | * | - | - | ** | ** | * | * | 7 |
| Preau et al. 2007 | * | - | - | ** | ** | * | * | 7 |
| Remor 2003 | * | - | - | ** | ** | * | * | 7 |
| Rueda et al. 2011 | * | - | - | ** | ** | * | * | 7 |
| Schnall et al. 2017 | - | - | - | ** | ** | * | * | 6 |
| Stasinopoulou et al. 2010 | * | - | - | ** | ** | * | * | 7 |
| Torres et al. 2018 | ** | * | - | ** | ** | * | * | 9 |
| Tran 2012 | * | * | - | ** | ** | * | * | 8 |
| Tran et al. 2012 | * | * | - | ** | ** | * | * | 8 |
| Uchechukwu et al. 2020 | * | - | * | ** | ** | * | * | 8 |
| Venturini et al. 2017 | * | - | - | ** | ** | * | * | 7 |
| Worthington & Krentz 2005 | * | - | - | ** | ** | * | * | 7 |
| Zuñiga et al. 2011 | * | - | - | ** | ** | * | * | 7 |
| Ahmed et al. (2011)a: Ahmed, Saqlain, Akhtar, et al. 2021; Ahmed et al. (2011)b: Ahmed, Saqlain, Bashir, et al. 2021  Note: - = not adequately assessed; high quality (9-8 stars total); moderate quality (7-5 stars total); low quality (> 4 stars total) | | | | | | | | |
